# Supplementary material for: Gonococcal OMV-delivered PorB induces epithelial cell mitophagy
Source: Nat Commun. 2024 Feb 23;15:1669. doi: 10.1038/s41467-024-45961-1 (PMC10891091; doi:10.1038/s41467-024-45961-1)

Gonococcal OMV-delivered PorB induces epithelial cell mitophagy

Shuai Gao, Lingyu Gao, Dailin Yuan, Xu'ai Li, Stijn van der Veen*

*E-mail: stijnvanderveen@zju.edu.cn

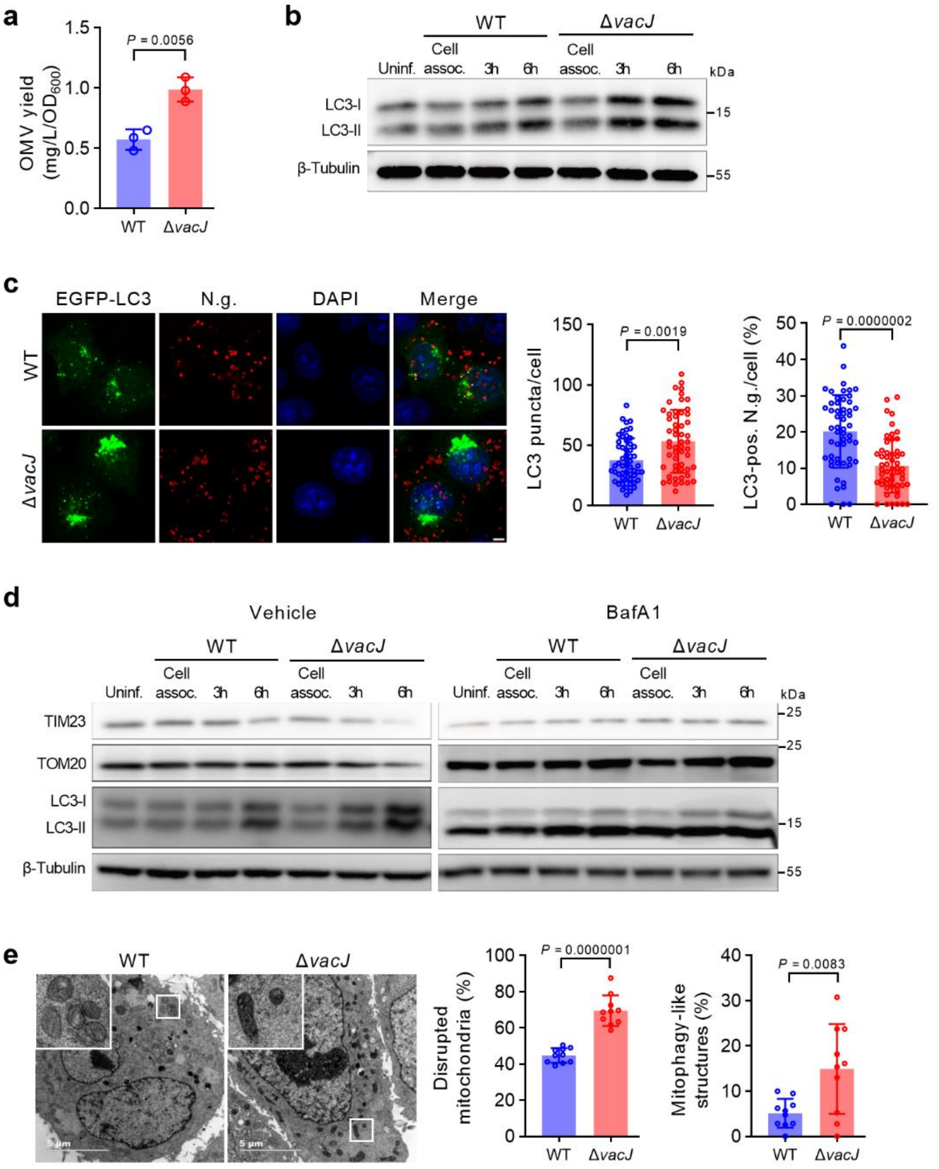

Supplementary Fig. 1 | The gonococcal Δ*vacJ* mutant shows enhanced OMV secretion and increased induction of mitophagy. **a** Quantification of OMV yields based on total proteins after OMV purification shows enhanced OMV yields obtained for the *Neisseria gonorrhoeae* Δ*vacJ* mutant. Data are mean ± s.d.; *n* = 3 independent experiments, unpaired two-tailed *t*-test. **b** LC3 Western blots after gentamicin protection assays show enhanced LC3 accumulation for the Δ*vacJ* mutant. **c** Microscopy images showing increased accumulation of total LC3 puncta for the Δ*vacJ* mutant after a one-hour challenge of HeLa cells, but reduced colocalization between the Δ*vacJ* mutant and LC3 puncta. Scale bar, 5 µm. Data are mean ± s.d.; *n* = 54 cells from 3 independent experiments, two-tailed Mann-Whitney test for quantification of LC3 puncta, unpaired two-tailed *t*-test for quantification of colocalized LC3 puncta. **d** Western blots of LC3 and mitochondrial marker proteins TOM20 and TIM23 after gentamicin protection assays with vehicle control- or BafA1-pretreated HeLa cells show enhanced mitochondrial degradation and autophagy flux for the Δ*vacJ* mutant. **e**

TEM images showing that a challenge of HeLa cells with *N. gonorrhoeae* results in increased mitochondrial disruption and mitochondrial capture in mitophagy-like structures for the Δ*vacJ* mutant (one hour after addition of gentamicin). Data are mean ± s.d.; *n* = 10 cells from 2 independent experiments, unpaired two-tailed *t*-test. Western blots in **b**, **d** are representative of 3 independent experiments. Source data are provided as a Source Data file.

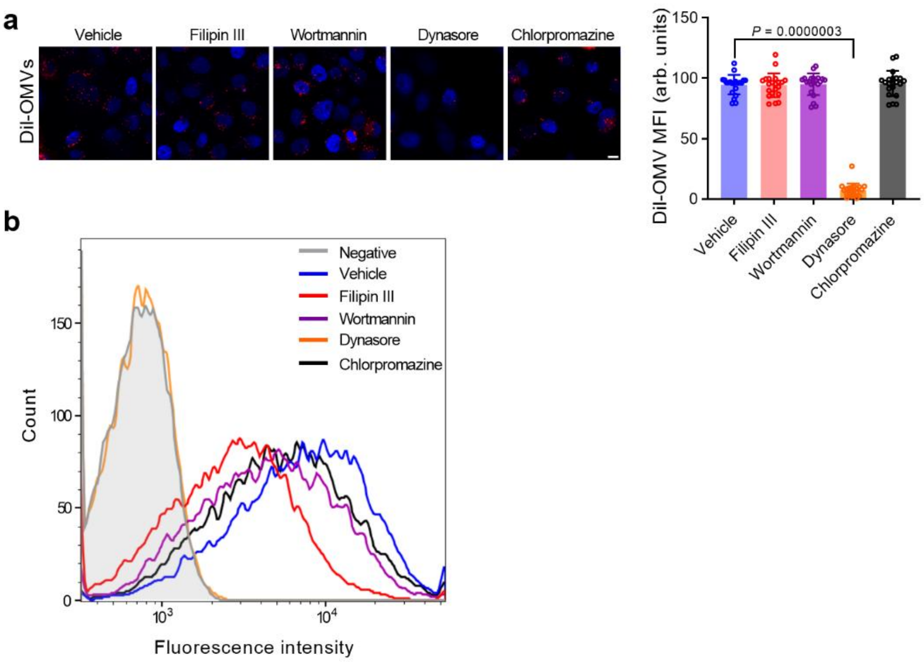

Supplementary Fig. 2 | Gonococcal OMV endocytosis in epithelial cells is dynamin dependent. a Microscopy images showing OMV endocytosis of HeLa cells is inhibited by the dynamin inhibitor Dynasore, and not by inhibitors of caveolin/lipid rafts (filipin III), macropinocytosis (wortmannin) or clathrin (chlorpromazine). Scale bar, 5 µm. Data are mean ± s.d.; $n = 21$ cells from 3 independent experiments, Kruskal-Wallis with posthoc Dunn test. **b** Flow cytometry analysis of Dil-OMV fluorescence intensity after endocytosis of HeLa cells showing endocytosis is dynamin-dependent. Source data are provided as a Source Data file.

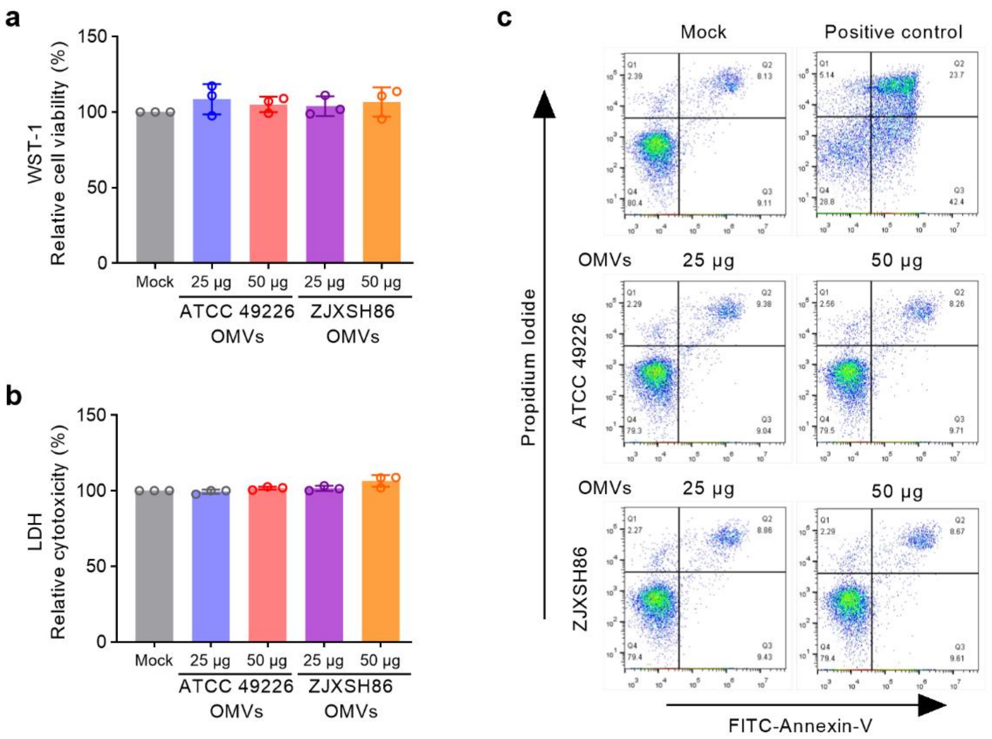

Supplementary Fig. 3 | Gonococcal OMVs do not affect cellular viability or induce cellular apoptosis. a Determination of HeLa cell viability with the WST-1 assay shows that OMV endocytosis does not affect cellular viability. Data are mean ± s.d.; n = 3 independent experiments. **b** Determination of OMV cytotoxicity with the LDH assay shows that OMVs do not display cytotoxicity after endocytosis of HeLa cells. Data are mean ± s.d.; n = 3 independent experiments. **c** Flow cytometry analysis of HeLa cells to detect for apoptosis using FITC-Annexin V/propidium iodide shows that OMV endocytosis does not induce apoptosis. Source data are provided as a Source Data file.

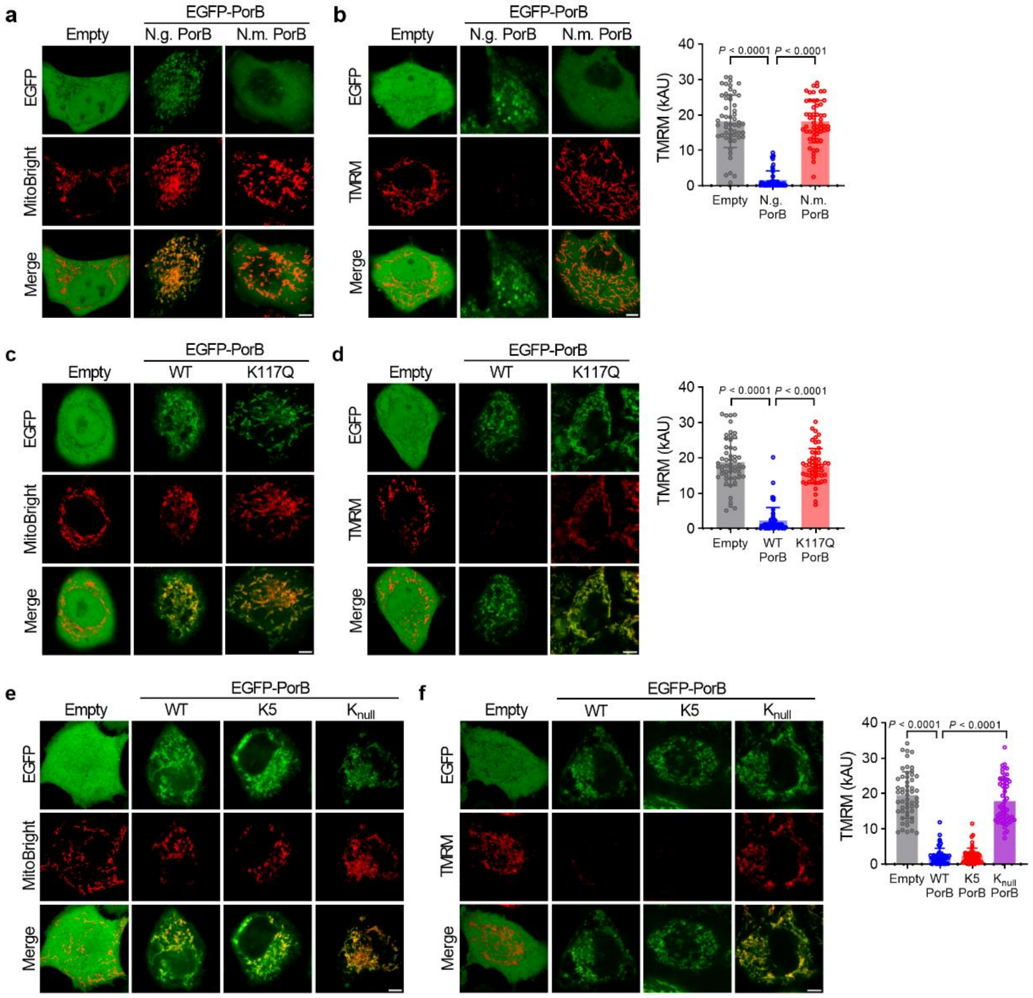

Supplementary Fig. 4 | Gonococcal PorB targets mitochondria and dissipates the mitochondrial membrane potential. a Representative microscopy images showing gonococcal PorB, but not PorB from *Neisseria mucosa*, localizes at HeLa cell mitochondria. Scale bar, 5 µm. **b** Representative microscopy images showing gonococcal PorB, but not PorB from *N. mucosa*, dissipates the mitochondrial membrane potential (MMP). Scale bar, 5 µm. Data are mean ± s.d.; $n = 54$ cells, Kruskal-Wallis with posthoc Dunn test. **c** Representative microscopy images showing that the gonococcal PorB K117Q mutant localizes at HeLa cell mitochondria. Scale bar, 5 µm. **d** Representative microscopy images showing that the gonococcal PorB K117Q mutant is unable to dissipate MMP. Scale bar, 5 µm. Data are mean ± s.d.; $n = 54$ cells, Kruskal-Wallis with posthoc Dunn test. **e** Representative microscopy images showing that the gonococcal PorB K5 (only retaining the 5 lysines associated with ATP binding in the PorB channel) and K_{null} (all lysines replaced by glutamines) mutants localize at HeLa cell mitochondria. Scale bar, 5 µm. **f** Representative microscopy images showing that gonococcal PorB K5 dissipates MMP, while PorB K_{null} lost this ability. Scale bar, 5 µm. Data are mean ± s.d.; $n = 54$ cells, Kruskal-Wallis with posthoc Dunn test. Images in **a**, **c**, **e** are representative of 3 independent experiments.Cells in **b**, **d**, **f** are from 3 independent experiments. $P < 10^{-15}$ for all reported values. Source data are provided as a Source Data file.

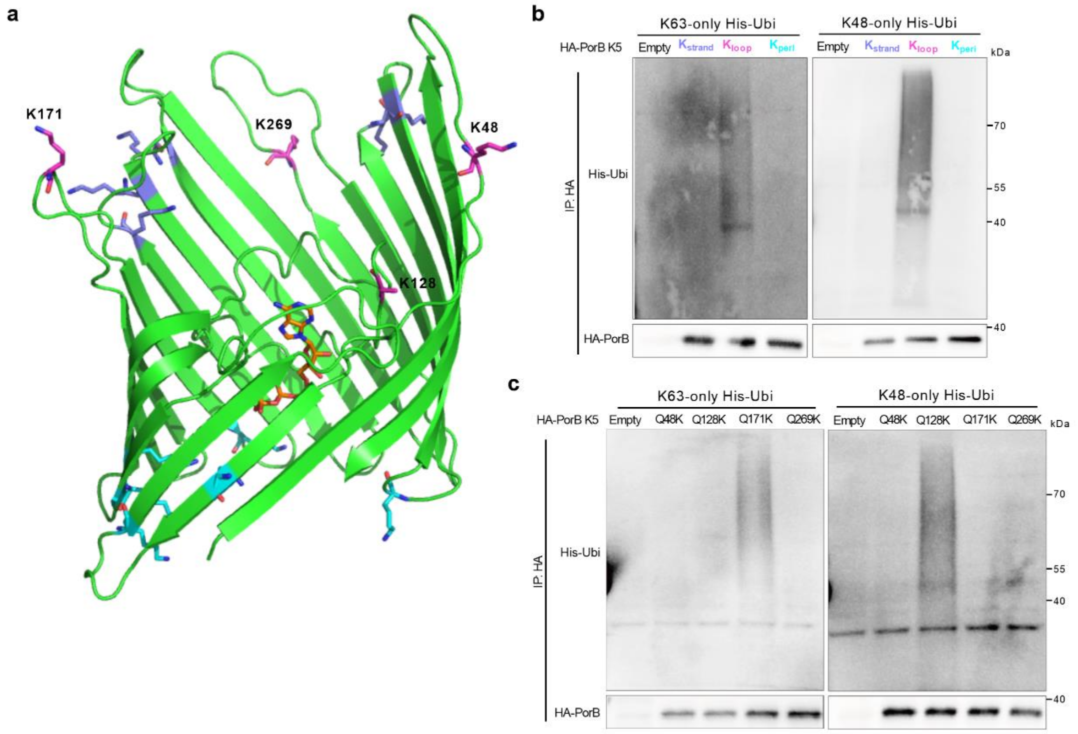

Supplementary Fig. 5 | Gonococcal PorB lysine 171 is decorated with K63-linked polyubiquitin. a Side-view of the gonococcal PorB structure (pdb entry 4AUI) with lysine residues retained by the PorB K_{strand} mutant displayed in purple, lysine residues retained by the PorB K_{loop} mutant displayed in magenta, and lysine residues retained by the PorB K_{peri} mutant displayed in blue. **b** Western blots after immunoprecipitation of HA-PorB K5 (only retaining the five lysine residues associated with ATP binding in the PorB channel)-derivatives K_{strand}, K_{loop}, and K_{peri} from HeLa cells show co-immunoprecipitation of both ubiquitin that can only form K63-linked or only K48-linked polyubiquitin for the PorB K_{loop} mutant. **c** Western blots after immunoprecipitation of HA-PorB K5-derivatives Q48K, Q128K, Q171K, and Q269K show co-immunoprecipitation of K63-linked polyubiquitin for the PorB K5-Q171K mutant and co-immunoprecipitation of K48-linked polyubiquitin for the PorB K5-Q128K mutant. Western blots in **b**, **c** are representative of 3 independent experiments.

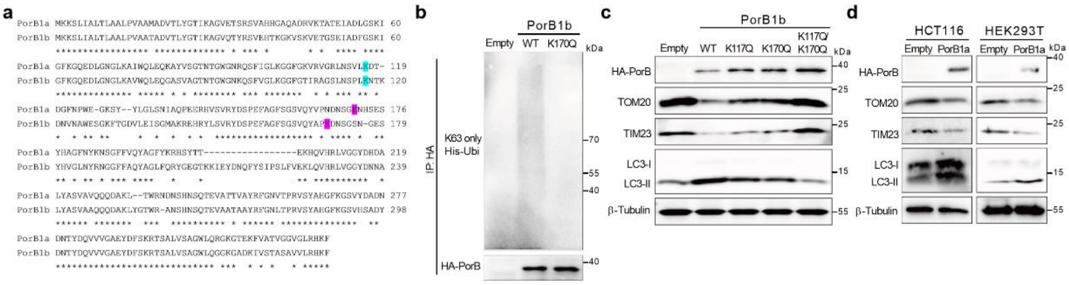

Supplementary Fig. 6 | Gonococcal PorB-induced mitophagy is not restricted to specific PorB type or epithelial cell types. a Amino acid sequence alignment of PorB1a from *Neisseria gonorrhoeae* strain ATCC 49226 and PorB1b from strain ZJXSH86, which share 70% sequence identity. PorB lysine residue 117 is highlighted in blue, and PorB1a lysine 171 and PorB1b lysine 170 are highlighted in magenta. **b** Western blots after immunoprecipitation of HA-PorB1b WT and the K170Q mutant from HeLa cells show that co-immunoprecipitation of ubiquitin that can only form K63-linked polyubiquitin is dependent on PorB1b lysine residue 170. **c** Western blots showing PorB1b-induced accumulation of LC3 and degradation of TOM20 and TIM23 in HeLa cells is dependent on PorB1b lysines 117 and 170, with degradation fully inhibited for a PorB1b K117Q/K170Q double mutant. **d** Western blots showing PorB1a-induced accumulation of LC3 and degradation of TOM20 and TIM23 in HEK293T and HCT116 cells. Western blots in **b**, **c**, **d** are representative of 3 independent experiments.

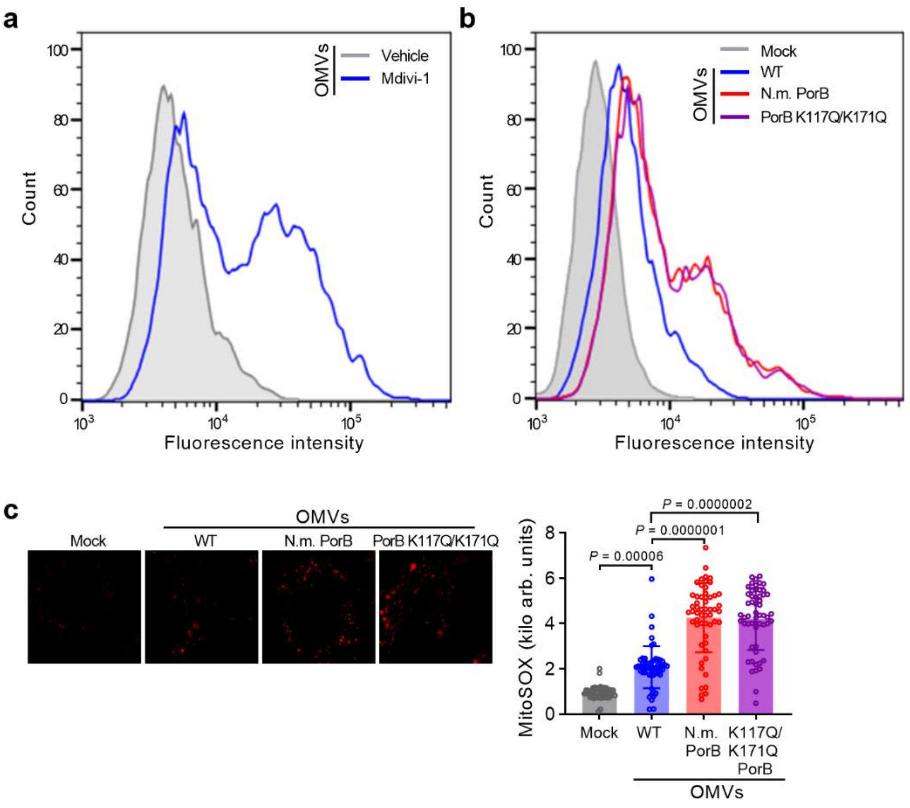

Supplementary Fig. 7 | Gonococcal OMVs induce mitophagy to reduce generation of mitochondrial ROS. a Flow cytometry analysis of fluorescence intensity of the ROS-sensitive MitoSOX probe after HeLa cell endocytosis of gonococcal OMVs shows that pretreatment with mitophagy inhibitor Mdivi-1 prevents OMV-induced reduction of mitochondrial ROS levels. **b** Flow cytometry analysis of fluorescence intensity of MitoSOX shows that OMVs expressing PorB K117Q/K171Q or PorB from *Neisseria mucosa*, which are unable to induce mitophagy, are not able to reduce mitochondrial ROS levels after HeLa cell endocytosis. **c** Live-cell microscopy images of ROS levels in OMV-stimulated HeLa cells show that OMVs expressing gonococcal PorB K117Q/K171Q or PorB from *N. mucosa* are unable to reduce mitochondrial ROS levels after HeLa cell endocytosis. Scale bar, 5 µm. Data are mean ± s.d.; $n = 55$ cells from 3 independent experiments, Kruskal-Wallis with posthoc Dunn test. Source data are provided as a Source Data file.

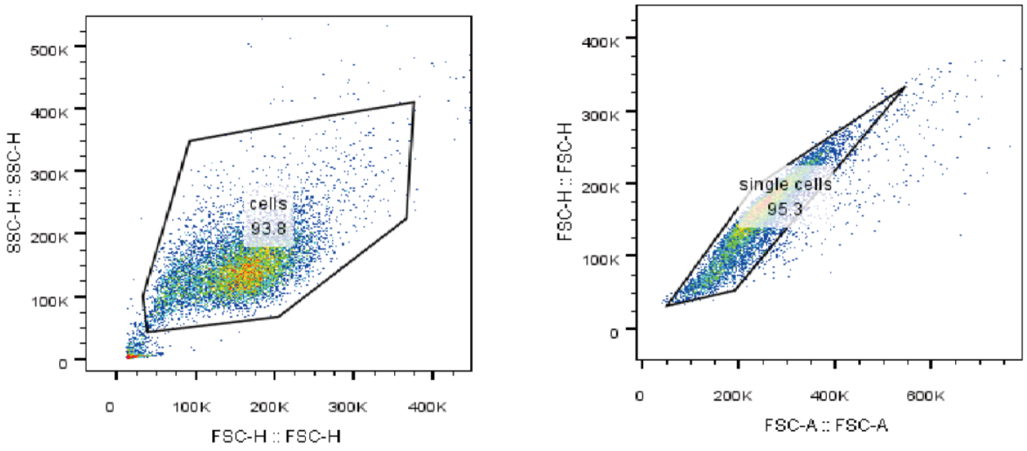

Supplementary Fig. 8 | Gating strategy. Representative plots showing selection of cells based on forward scatter height (FSC-H) and side scatter height (SSC-H), and single cells were selected based on FSC-H and forward scatter area (FSC-A) to exclude doublets.

Supplementary Table 1 | Primers used in this study.

Primer name	Primer sequence 5'-3'
vacJ-upF	GCGAGCTCAGTTCTGCAATGGTTCTGG
vacJ-upR	GCGAATTCGATTTCAGCCAGTCTTTGA
vacJ-downF-1	GCGAATTCAGCCATGCCGTCTGAAAAT
vacJ-downR-1	GCTCTAGAGTAAACGGCAGTCCGTAGT
vacJ-downF-2	GCAGATCTAGCCATGCCGTCTGAAAAT
vacJ-downR-2	GCGTCGACGTAAACGGCAGTCCGTAGT
ldcA-UF	GCGAGCTCCAGACGGCATTGTTCTCCC
ldcA-UR	GCTCTAGAAATTTCCCTTTCATACGGT
ldcA-DF	GCGGATCCACCCGCAAACGGACAAAATG
ldcA-DR	GCCTGCAGCTGCTGGCAATCTTTGTCG
coxII-q-F	GCGCAAGTAGGTCTACAAG
coxII-q-R	GGCAGGATAGTTCAGACGG
human RPL13A-F	CGCCCTACGACAAGAAAAAG
humanRPL13A-R	CCGTAGCCTCATGAGCTGTT
porB-F	GCGAATTCATGAAAAAATCCCTGATTGCCC
porB-R	GCGGATCCTTAGAATTTGTGGCGCAGAC
porB(2)-F	GCGAATTCATGAAAAAATCCCTGATTGCCC
porB(2)-R	GCGGATCCTTAGAATTTGTGGCGCAGAA
porB-K117Q-F	TTGAACAGCGTCCTGCAAGACACCGACGGCTTCAAT
porB-K117Q-R	GAAGCCGTCGGTGTCTTGCAGGACGCTGTTCAAACG
PorB-Q48K-F	GCGGATCGCGTTAAAACCGCTACCGAAATCGCT
PorB-Q48K-R	TTCGGTAGCGGTTTTAACGCGATCCGCCTGAGC
PorB-Q128K-F:	CCTTGGGAGGGTAAAAGCTACTATTTGGGTTTA
PorB-Q128K-R	CAAATAGTAGCTTTTACCCTCCCAAGGATTGAA
PorB-Q171K-F	GACAATTCGGGCAAAAATCACAGCGAATCTTAC
PorB-Q171K-R	TTCGCTGTGATTTTTGCCCGAATTGTCGTTAGG
PorB-Q269K-F:	GCCCACGGCTTCAAAGGTTCGGTTTATGATGCA
PorB-Q269K-R	ATAAACCGAACCTTTGAAGCCGTGGGCGTAAGA
PorB-K128Q-F	CCTTGGGAGGGTCAAAGCTACTATTTGGGTTTA
PorB-K128Q-R	CAAATAGTAGCTTTGACCCTCCCAAGGATTGAA
PorB-K171Q-F	GACAATTCGGGCCAAAATCACAGCGAATCTTAC
PorB-K171Q-R	TTCGCTGTGATTTTGGCCCGAATTGTCGTTAGG
PorB(2)-K117Q-F	CTGAACAGCCCCCTGCAAAACACC
PorB(2)-K117Q-R	GTTGTCCTTGGTGTTTTGCAGGGG
PorB(2)-K170Q-F	GTACAATACGCACCTCAAGATAAT
PorB(2)-K170Q-R	TGAGCCTGAATTATCTTGAGGTGC
dus-mucosa-F	ATGCCGTCTGAAATGCCGTCTGAAGGTTTGCAA
dus-mucosa-R	TTCAGACGGCATTTCAGACGGCATTGACGGAAG
dus-porB-F	ATGCCGTCTGAAGAATTCCCAATTCGTACGCT
dus-porB-R	TTCAGACGGCATAAGCTTTGACGGAAGGCAAC

Supplementary Figures: Uncropped Western blots

Supplementary Figure 1b

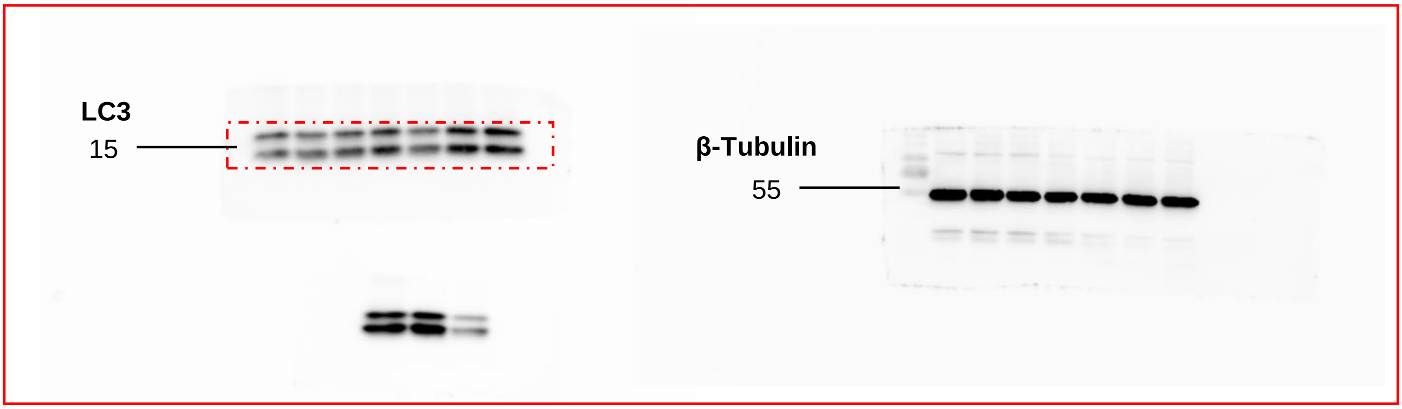

Supplementary Figure 1d

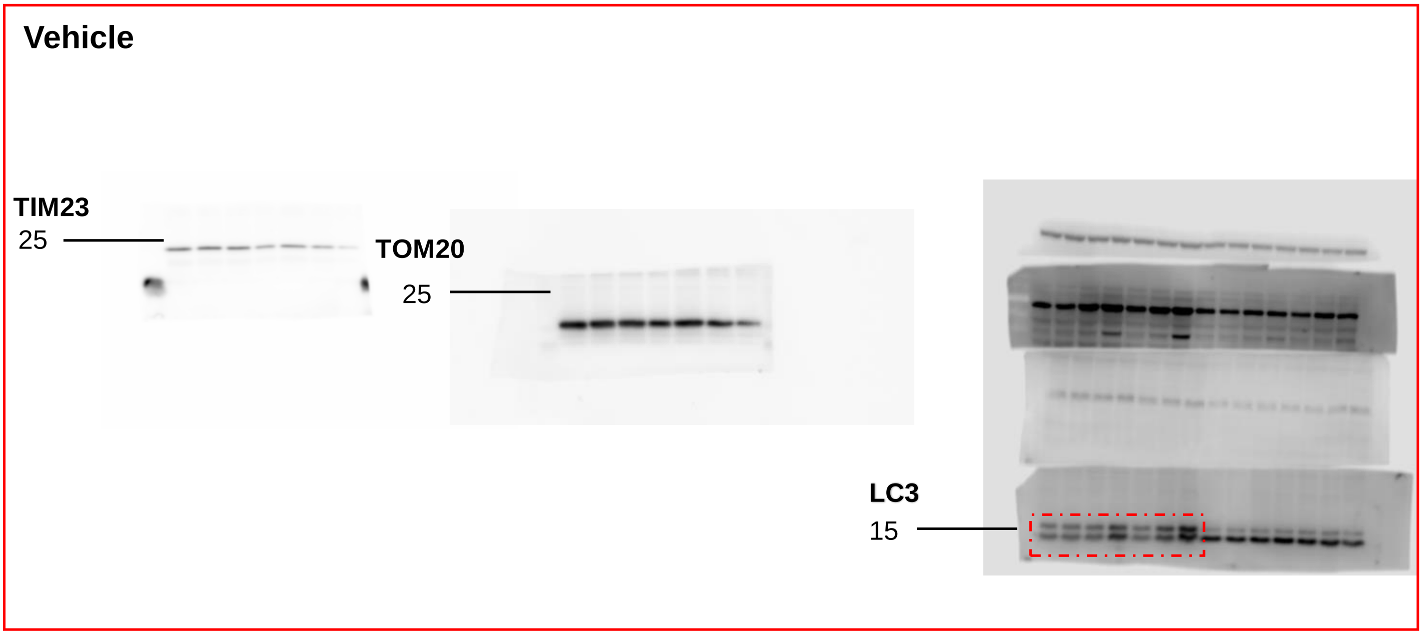

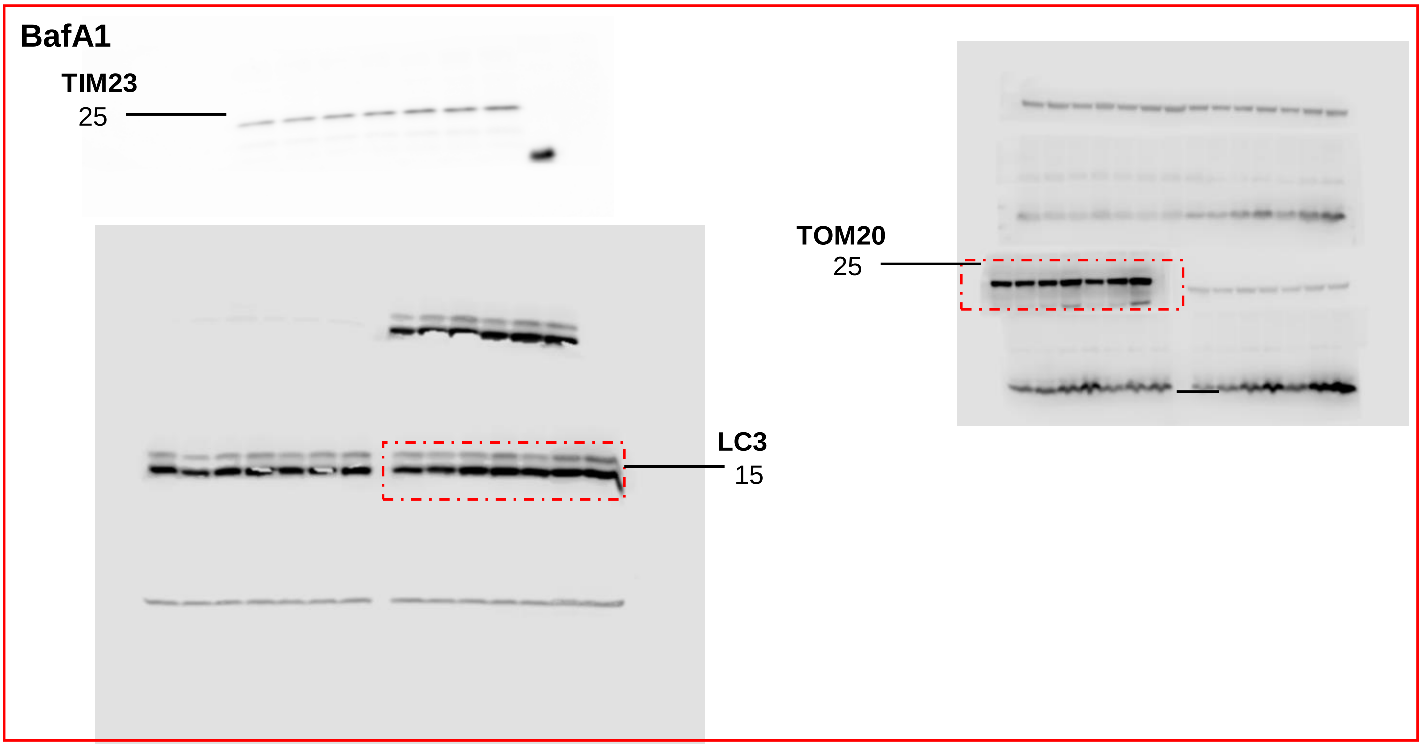

Supplementary Figure 1d

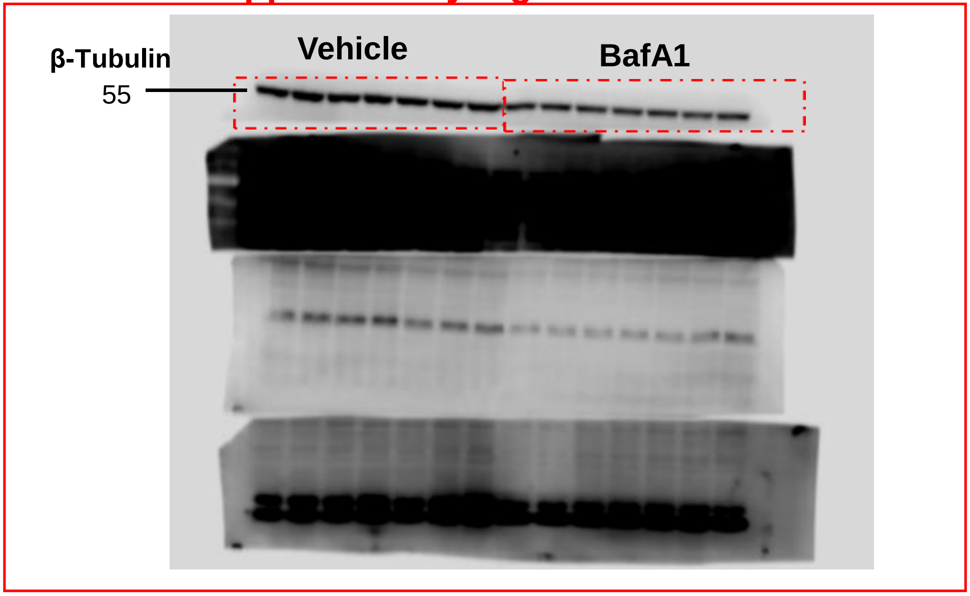

Supplementary Figure 5b

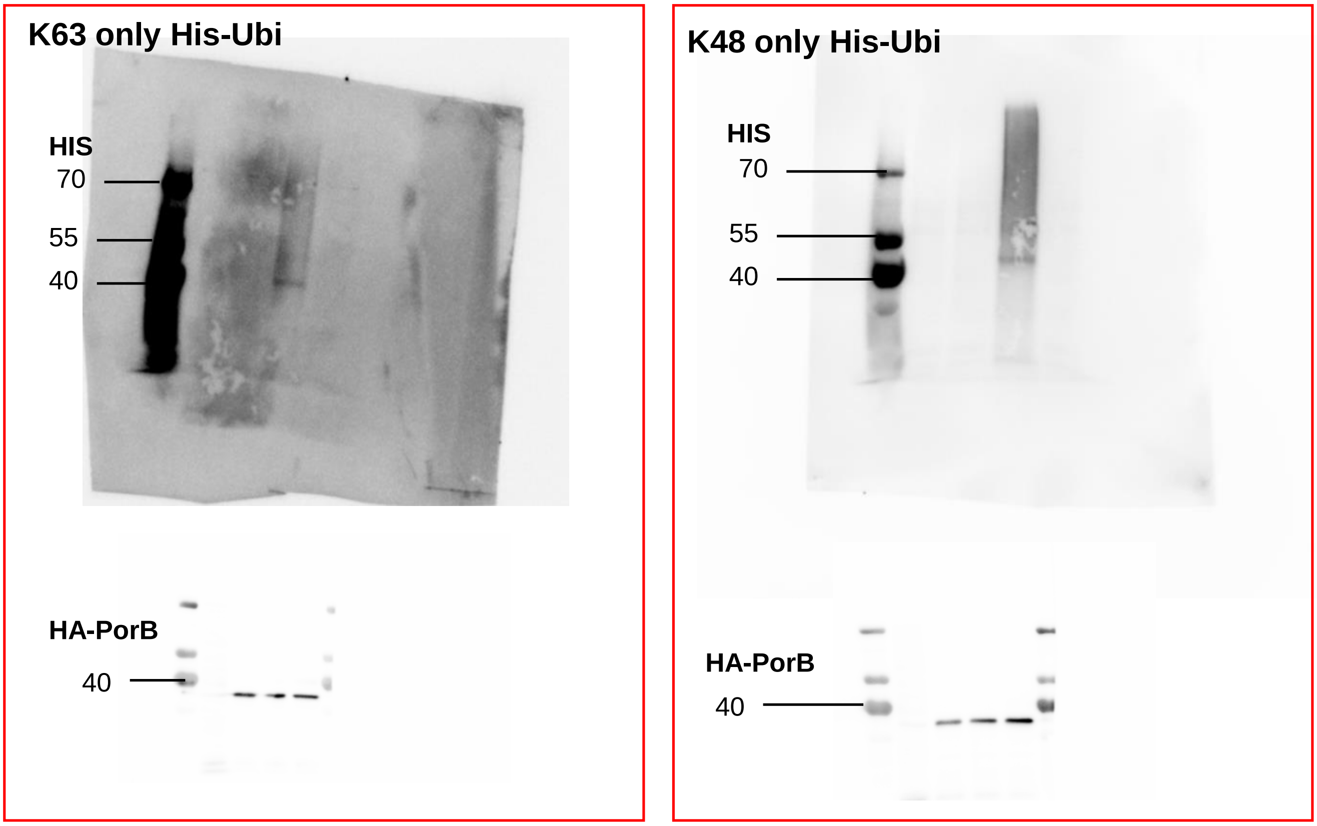

Supplementary Figure 5c

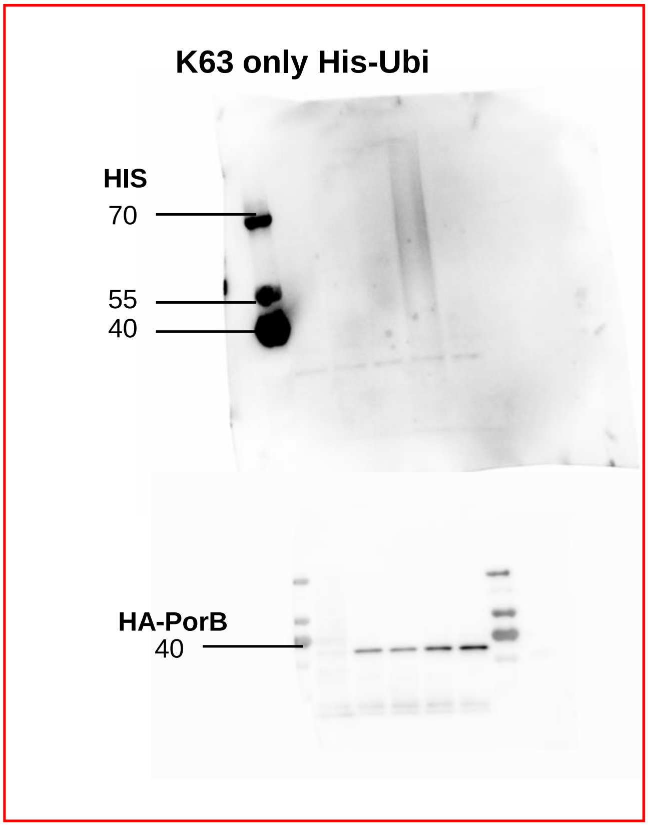

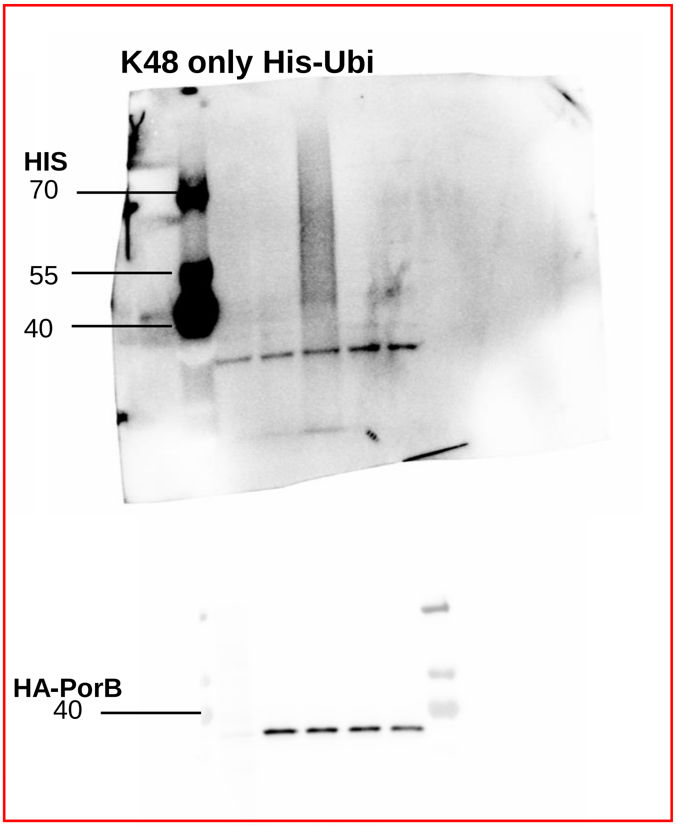

Supplementary Figure 6b

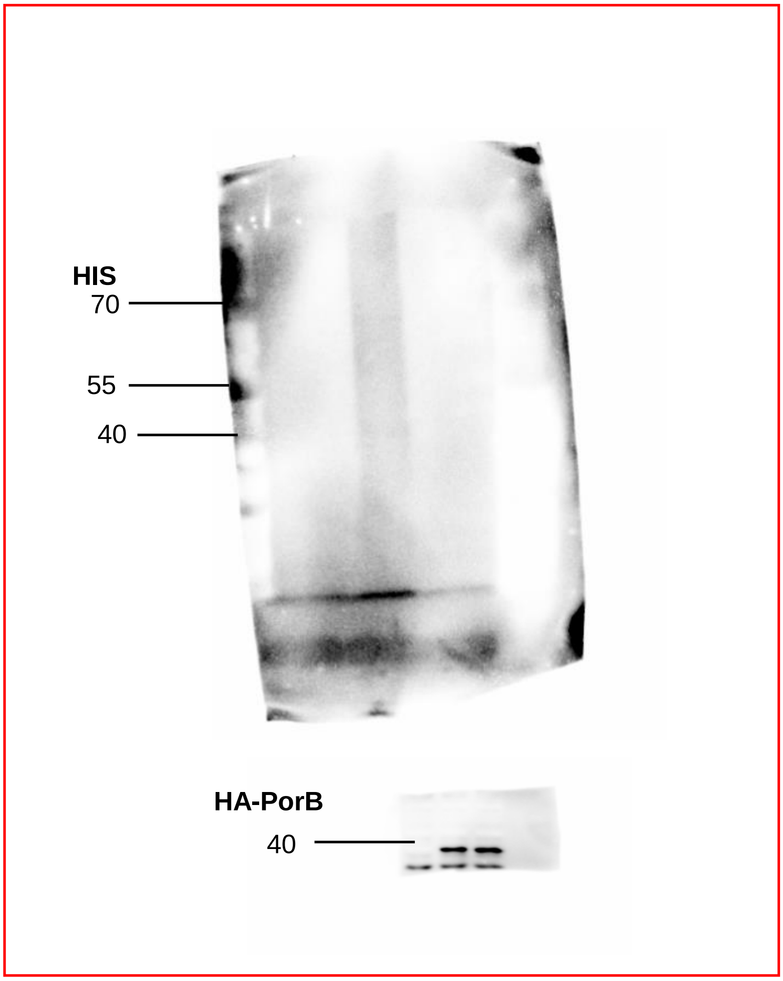

Supplementary Figure 6c

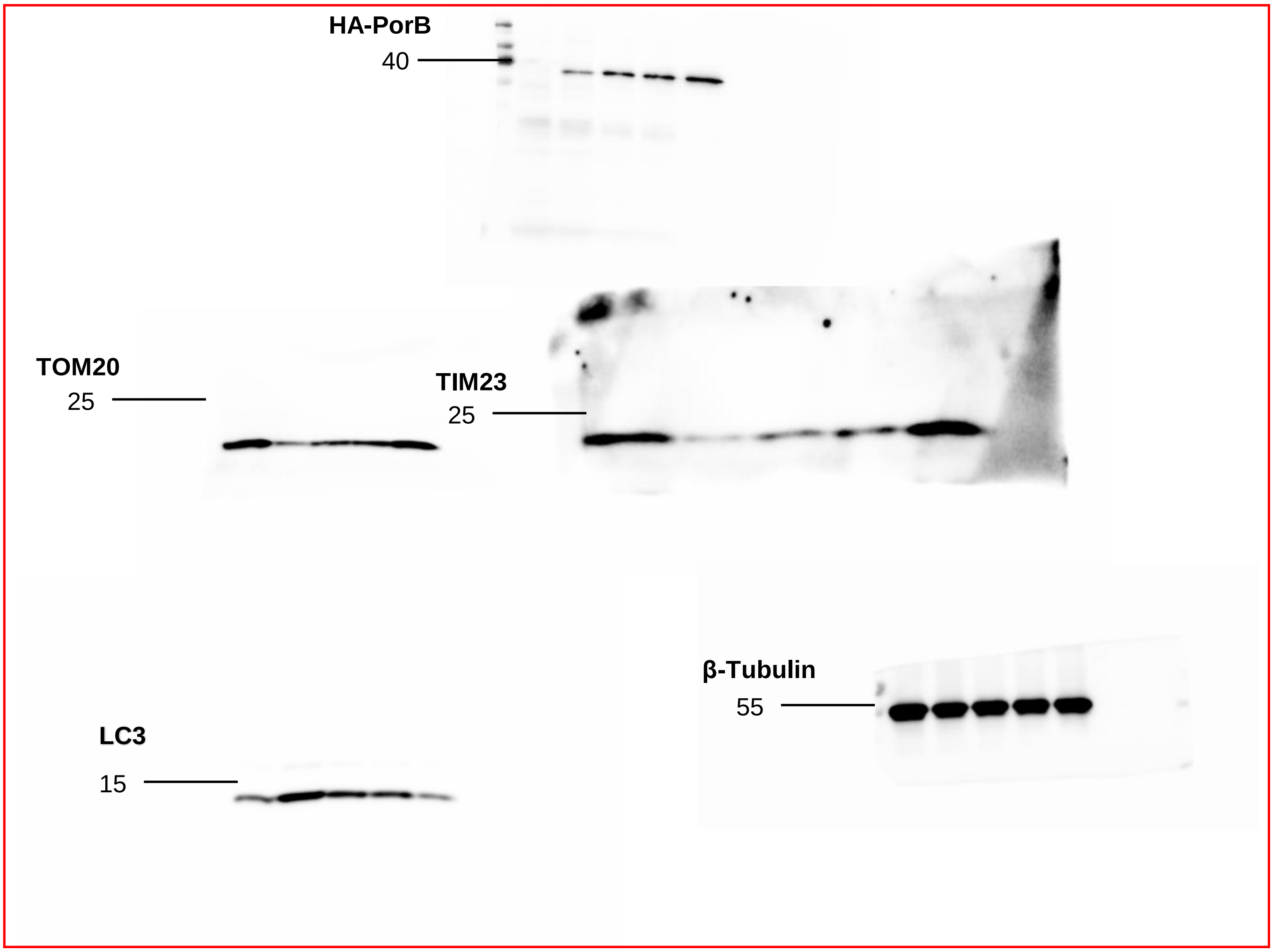

Supplementary Figure 6d

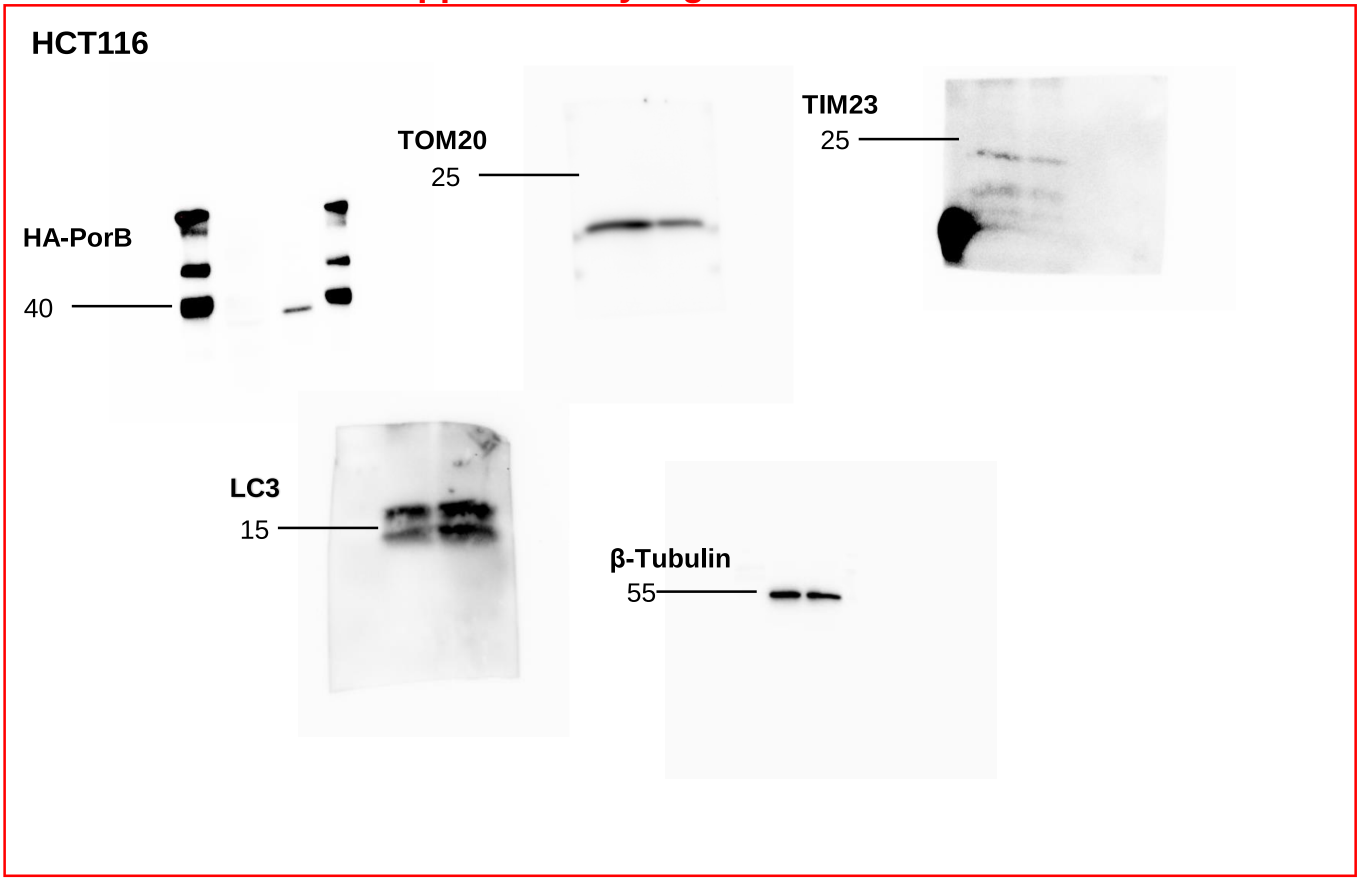

Supplementary Figure 6d

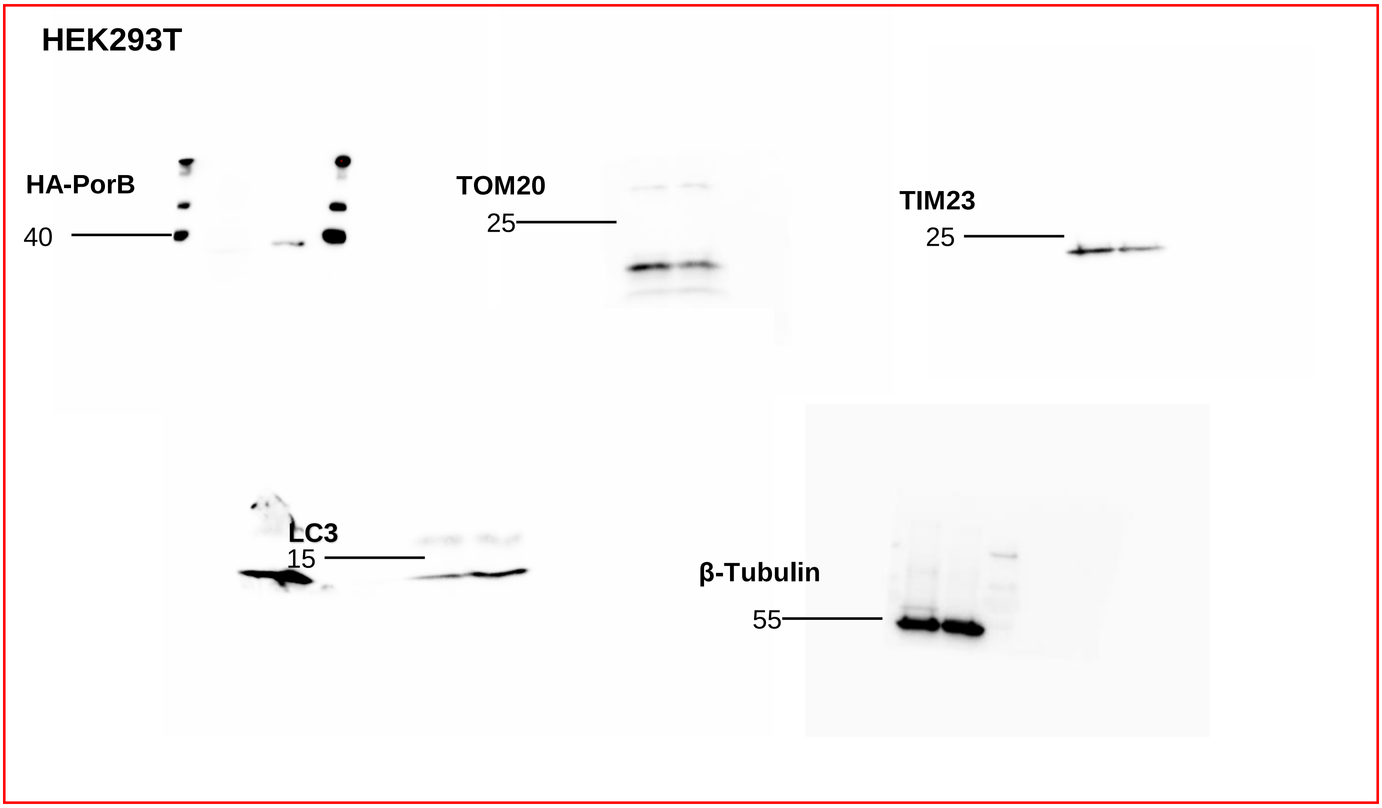

Supplement: Supplementary file 1 — Supplementary Information [file 41467_2024_45961_MOESM1_ESM.pdf]
